# How to write a successful grant or fellowship application

**DOI:** 10.1136/practneurol-2015-001206

**Published:** 2015-09-18

**Authors:** Masud Husain

**Keywords:** CLINICAL NEUROLOGY

Successful grant writing takes careful thought as well as considerable skill. Experienced investigators appreciate just how much work and background development are required. However, those new to the ‘game’ are sometimes under the misconception that if they have a good research idea or it is clinically important, they are bound to succeed. ‘Do good science and the rest will follow!’ Unfortunately, this just is not true.

Successful grant writers appreciate three important points:

1. Don't take anything for granted!
  Even if you have a superb track record and great ideas that could fundamentally change a field, this is not an excuse for cutting corners and dashing off a poorly thought-out submission. Grants require care. Sloppiness is obvious.
2. Put your energy into a few, well-crafted submissions
  It does nothing for your morale, or reputation, to keep resubmitting poor applications that fail. Reviewers and panel members have long memories.
3. The competition is tough
  Success rates are low for most schemes, often less than 20%, sometimes even down to 10%. And your competition is stiff. It includes some of the finest people of your generation, your most able contemporaries. So, to stand a chance of success you have to give grant writing—and your competition—the respect it deserves.

When you submit an application, it is likely first to be screened by administrative staff to ensure that it fulfils the basic requirements. Some grants fall at this very initial hurdle. In some schemes, there might then be a triage or selection procedure, to filter out the applications that are unlikely to succeed, so your submission might not get any further than this. Then applications are sent for peer review, often from international experts who might not know you and sometimes by people who might not even have much expertise in your particular subspecialty of research. When these reviews return, a panel of experts put together by the funding agency, will assess your application with the comments of the reviewers to help them. In some schemes, you might be asked to come to interview to defend your application in front of the panel. For others, the decision is made directly by the panel. In both cases, one or two members of the panel will be asked to speak about your application, perhaps for only a couple of minutes.

This is the critical moment where your grant stands or falls. The panel members who lead the discussion on your application have to be persuaded that it is worth pushing for. If they are to be your advocates, they have to appreciate the quality of your proposed research programme, why it is important and why it deserves support. This just will not happen unless your submission has been written clearly and in a compelling manner. Remember that panel members come from diverse backgrounds and there may not be anyone who is an expert in your area of research. For example, your work might be on molecular neuroscience, but the panel member leading the discussion might be someone who specialises in neuroimaging. They have to ‘get’ your proposal if they are going to convince the rest of the panel (often consisting of people from fields other than neurology or neuroscience) that this is worth supporting.

## Key reasons for success and failure

In my experience—as an applicant, reviewer and grant panel member—the key reasons for success include the points in box 1.

Box 1 **Ten top reasons for success**

The application

1. Fits the Call or Fellowship scheme well
2. Is carefully crafted and polished over time, improved by colleague feedback
3. Is timely, pertinent and asks good—even crucial—questions for the field
4. Is hypothesis driven and intellectually stimulating
5. Is clear, readable and intelligible
6. Shows that you are passionate about this topic
7. Provides pilot data and follows credibly from established findings
8. Justifies sample sizes with power calculations
9. Is to performed in a centre of excellence
10. Builds on your track record

I will discuss some of these in more detail later but first it is also worth considering some major reasons for failure. For me, these include the following:

1. The submission is unclear, written in haste or just poorly put together
  Remember that it has to be easy to read and comprehend by reviewers who are extremely busy. You might think that the text you have laboured over for hours will be evaluated with great care. On many occasions, however, your applications will be speed read, perhaps in less than half an hour, in a setting where the reviewer is vulnerable to being distracted—on a train or a flight, at home with children running around or in a busy office where there is always someone knocking on the door. If the text is not crystal clear, what you think—or assume—is obvious will be missed. This is one reason why it is essential to get your submission read by colleagues long before you submit. Unfortunately, most people leave this to the last minute when it is usually too late.
2. No hypotheses or poorly articulated ones
  This is a common reason for being shot down at a grant panel when your application is discussed. It is surprising how many submissions just do not have explicitly stated hypotheses. Goals and aims are not the same as saying what you are testing. When you do state your hypotheses, they have to be signposted well.
3. Inadequate track record or expertise in this area
  Collaborations with experts can help to get around this criticism, so think about approaching collaborators within or outside your institution early and get then to write a letter of support. In addition, try to get as much leverage as you can from what you have already published.
4. Far too ambitious
  A common criticism is that the applicant could not achieve all that is proposed in the time available for the grant. There is a difficult balance to achieve between promising enough and far too much. The key point is that all that is proposed must be feasible within the time scale. Your reviewers have lots of experience and they know what is practical.
5. Great ideas but no preliminary data
  You have to convince reviewers that your proposal will work. If this is a completely new approach, no matter how exciting it might be, the grant panel will require some evidence for its credibility. When in doubt get as much pilot data as possible before submitting.
6. Incremental research, not a step change
  This is a difficult issue. If the proposal seems too conservative and incremental, it might be rejected because it is not exciting enough and will not deliver sufficiently novel findings. On the other hand, if it is too risky and ‘blue-skies’ it might be considered a gamble. The best proposals incorporate a combination of both elements: build incrementally on previous work—either by you or by the group you hope to join—but also think about proposing a riskier, step change element. Remember though that blue-skies ideas are good for only some parts of a submission but not for all of it.

## Before you start

It is important that even before you start putting pen to paper you are clear why you are writing this application. Motivation really does matter. Ideally, you will be applying because you have some great ideas that you have thought about carefully and really want to test. Furthermore, you have the energy and enthusiasm to pursue this programme of work. In short, you should be motivated to make this work. You really should not be writing an application just because your boss thinks it might be a good idea or because there is a new Call which is vaguely in your area or simply because you feel that it is worth a ‘punt’. Grant writing takes a lot of time and energy and by the end of an application most people are drained. So think carefully about your motivations before you even start. If you are not convinced or driven by this, it is unlikely to succeed.

## Does it fit the scheme?

Be careful about reading the specific requirements of the Call or Fellowship scheme. Contact the funding agency, if necessary. Don't be frightened to discuss the proposal you have in mind with one of the administrators there. They often have been in science themselves and are very helpful if approached appropriately. Find out from them and your colleagues about what sort of proposals have been successful before. Then check whether you would have institutional support for applying to this scheme. Don't surprise your boss by asking him or her to endorse your application at the last minute. Heads of departments do not like surprises! It is well worth finding out what the institution would be willing to commit to in terms of supporting you for this application and the implications that this might have for the future. Finally, re-read the instructions and be clear in your own mind that your proposal fits exactly what the scheme is designed for.

## What makes a good research question?

If you put this to leading researchers you are likely to get many different answers. So ask them! You will learn a lot from the process and from interacting with them. I think a good research question has to be *obviously good* to people from outside your research area. It has to be big enough for others to appreciate immediately why anyone should spend years of their life using large amounts of money from the public purse or charities to answer such a question. For many researchers, the question also has to be intellectually stimulating—if possible, thrilling! But at the same time a good research question has to be one which is likely to be answered within the time frame of the grant. It has to be a practical proposal, not something so grand that it would take decades to unlock.

## Don't rush it

**Figure 1:**
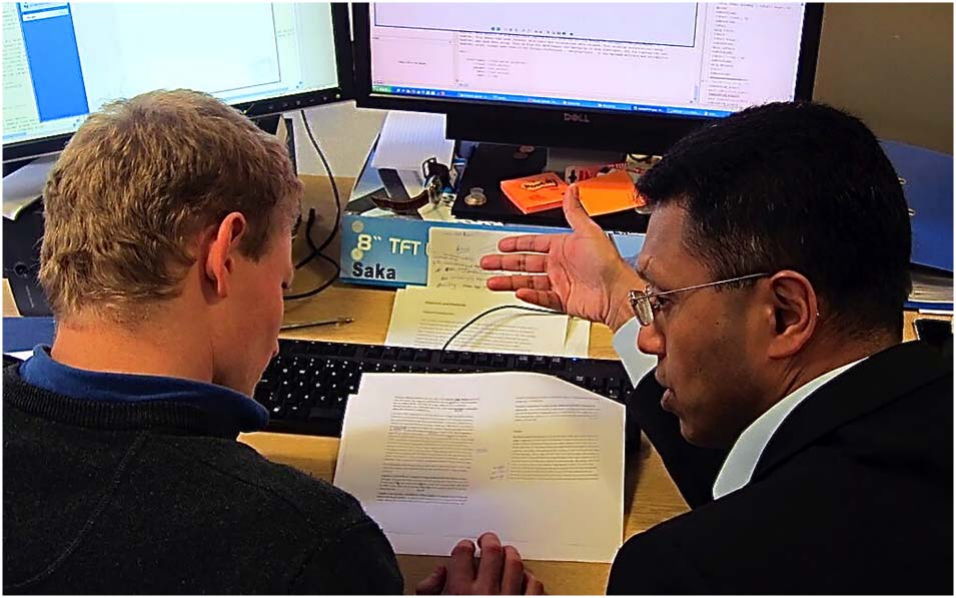
Don't be shy to ask for advice from more experienced colleagues.

Putting together a coherent and cohesive set of studies to answer your question is not easy. Ideally, before you start to write you should have assembled a plan of possible studies from which you need to cherry pick your best ideas for this particular application. You need time to get comments and feedback from colleagues to see whether they are convinced. This really does matter and the more experienced members of your department or your collaborators will be able to give you helpful advice (figure 1), provided you don't leave it until the last minute. It is vitally important to polish up an application properly so that everything is crystal clear and cohesive. Remember also that costings take time and may alter your proposal if it turns out that the sums you require would be well above what the funding body is prepared to offer. Full Economic Costing in the UK also adds to the final total and it is important to be aware for which grant giving bodies this might be a factor.

## Make it hypothesis driven

It is well worth jotting down what your hypotheses are, for your own sake. Most studies are vulnerable to the criticism that they are ‘exploratory’ and do not have specific hypotheses that are being tested. Even if your study is largely exploratory, make some explicit hypotheses about what you might be directly testing, based on proposal. It is absolutely essential that you write down clear hypotheses, unless the Call is specifically for an exploratory study, which is very rare. ‘Fishing expeditions’, no matter how good they might be, do not fare well with reviewers or grant panels. I often use direct questions incorporating the hypotheses I want to test as subheadings in an application. That way no one is in doubt about what the hypotheses are.

## Writing the document: put yourself in the reviewer's shoes

While you are writing imagine how you would feel reading this material. The narrative has to be absolutely clear and coherent, with a linear trajectory. No matter how scientific or clinically applied your submission, remember that you are telling a story that the reviewer has to get immediately. Moreover, as explained earlier, you have to appreciate that many reviewers and even panel members might not actually be in your field of expertise. For many schemes, particularly Fellowships, panel members will not be even in your general area, so they need to understand why your case is so compelling and important. This is why the overview and strategic vision must be both clear, as well as exciting. Unfortunately most are relatively dull! One way to improve this is to write the summary for lay people at an early stage. Unfortunately these usually do not receive enough attention and are scrambled together at the last minute. However, you would be surprised how often reviewers and panel members read these summaries first to get an idea of what you are trying to do. Polish these well! It is well worth the trouble.

The proposal also has to be intellectually exciting, even for the non-specialist.

Make the document easy to read in terms of its formatting. Avoid clutter, use figures and boxes wherever you can. Try not to cram the entire document with dense text. Signpost a new section clearly and consider stating the hypotheses for that section up front with bullet points so they stand out and will not be missed. Use lots of paragraphs to break up the text and to make it readable. Do not go over the word limit. Panel members become particularly irritated if this is evident. Above all, you don't want to hand them excuses to reject your application: because they do not understand it well enough, it just does not seem very exciting or you did not stick to the rules of the application.

## Sample sizes

All studies need to consider a justification for the sample size. This is an issue that can be relatively easy to address explicitly but you would be surprised how many applications do not include a power calculation. For some, applications you need to provide evidence you did this with a statistician. For others, you can do it yourself using free software (eg, G*Power at http://www.gpower.hhu.de). Sometimes it is not easy to provide a power calculation for certain types of research. If this is the case, you need to explain why and ideally refer to a previous study which successfully used a sample size like the one you propose in order to answer a related question.

## Potential weaknesses of the proposal

All proposals are vulnerable to criticism. Some reviewer might consider the study design to be inadequate to answer the question. Others might not be convinced that alternative explanations for predicted results have been considered. You can try to strengthen your proposal by considering these criticisms and building measures to counter them, for example, adding further controls or by considering more carefully the design and analyses. Think also about contingency plans and whether you should explicitly discuss these. If your entire programme of research depends upon an initial study being successful, you are vulnerable to the criticism that there is no plan should it fail. Again, this is where early reading by experienced colleagues might help you to head off the killer criticisms that a reviewer might raise. Don't be shy to ask for their help. They might, for example, advise you how to avoid a linear, sequential strategy by creating a more ‘parallel’ research proposal, with several different studies converging to answer a research question.

## Use figures and illustrations

Many submissions consist of endless pages of text. Figures help to break up the appearance and make the application more appealing to read. They also can be used to improve the clarity of your proposal, to illustrate concepts or add flesh to the text with a concrete example. Take care to write the figure legends well. These allow you to reiterate points made in the text; saying it differently increases the likelihood of a difficult point being understood. Figures are also essential if you want to show off pilot data. A plot of the findings has far greater impact than a line in the text, which is easy to miss.

## Impact

This is an important trend for many grant funders. Ask yourself what your big idea is. Are you going to fill a knowledge gap? Would anyone care? Is the proposal timely? And how would it make a difference if you were successful? The societal, economic, health, intellectual property and other impacts might be important in different ways for different grant funding bodies. Understandably, many researchers feel the pressure to make a case for their work being clinically important. However, it is important also to appreciate that there is no point in simply gesturing towards ‘translational’ research. If your application is not applied directly to a clinical problem, don't pretend. It annoys reviewers. Vision can nevertheless be displayed, in part, by spelling out the next steps, for example, ‘If we find this biomarker/gene/imaging signature, then we will proceed to developing clinical screening/mouse model/MRI protocol but the resources for this lie outside the current application’.

For some grants, it is extremely important to explain how potential beneficiaries might have the opportunity to benefit from your work. RCUK impact pages (http://www.rcuk.ac.uk/innovation/impacts/) offer helpful guides. Box 2 also gives some important reasons for poor impact in proposals. Dissemination and public engagement needs to be thought about early on. It is also important to think about how you might facilitate ‘knowledge exchange’ which ideally would be a two-way process, for example, between patient groups and your research group.

Box 2 **Characteristics of poor impact proposals**

- Lack of specificity on deliverables
  What exactly will you have achieved at the end of the grant? How might it affect people outside your field? What would be the wider impact?
- Lack of consideration of broader beneficiaries and stakeholders
  If your work has potential to have wider impact, don't be shy to mention these. It can sway the decision making of the grant panel.
- Proposal is too narrowly focussed
  Is the potential for impact too narrow? If so, how might you widen it?
- Too much focus on track record rather than what will be done
  Make sure you devote sufficient text to the details of the project and articulate the wider significance of this work.

## Don't give the panel excuses to reject

Finally, try not to hand the panel or reviewers any reasons for rejecting you. I have covered some reasons for panel irritation above. Some recurring themes in committee discussions include the points in box 3, which might serve as a useful list to consider—and reconsider—as you polish your application.

Box 3 **Reasons for rejection by grant panel**

1. No hypothesis
2. Far too incremental; not a ‘step change’
3. Dull, not sufficiently exciting
4. Great ideas but no pilot data
5. Great ideas but far too ambitious—impractical, given time and resources
6. Sample sizes not justified with power calculations
7. Proposal has too many potential weaknesses; insufficient controls
8. Does not have strong enough track record
9. Plans to work in a centre which has insufficient experience in this field
10. Unclear why this is interesting or what the impact of work would be

It should be obvious by now that writing a grant is a formidable endeavour. The low success rates mean that it is simply not worth putting together an application that has not been well thought-out or is assembled at the last minute. If you do have the motivation, I hope the advice distilled into this short article will offer a little assistance in getting you across the finishing line, with success.

Key points

- Successful grant writing takes time and care, so plan well ahead of the deadline.
- Proposals need to fit the call.
- They should be clear and exciting, but also feasible to complete within the time frame.
- Applications can be improved with advice from more experienced colleagues, so do ask for help.
- Ideally they should be hypothesis-driven, have supporting pilot data and justification of sample sizes.
- Their potential for impact on a scientific question, patient group or for Society needs to be clearly stated.

